# Overcoming Cancer Drug Resistance with Nanoparticle Strategies for Key Protein Inhibition

**DOI:** 10.3390/molecules29173994

**Published:** 2024-08-23

**Authors:** Hyeonji Yoo, Yeonjin Kim, Jinseong Kim, Hanhee Cho, Kwangmeyung Kim

**Affiliations:** Graduate School of Pharmaceutical Sciences, College of Pharmacy, Ewha Womans University, Seoul 03760, Republic of Korea; mary927@ewhain.net (H.Y.); duswls223@ewhain.net (Y.K.); jinseong@ewha.ac.kr (J.K.); ricky@ewha.ac.kr (H.C.)

**Keywords:** drug resistance, nanoparticle, drug delivery system, cancer therapy, targeted therapy

## Abstract

Drug resistance remains a critical barrier in cancer therapy, diminishing the effectiveness of chemotherapeutic, targeted, and immunotherapeutic agents. Overexpression of proteins such as B-cell lymphoma 2 (Bcl-2), inhibitor of apoptosis proteins (IAPs), protein kinase B (Akt), and P-glycoprotein (P-gp) in various cancers leads to resistance by inhibiting apoptosis, enhancing cell survival, and expelling drugs. Although several inhibitors targeting these proteins have been developed, their clinical use is often hampered by systemic toxicity, poor bioavailability, and resistance development. Nanoparticle-based drug delivery systems present a promising solution by improving drug solubility, stability, and targeted delivery. These systems leverage the Enhanced Permeation and Retention (EPR) effect to accumulate in tumor tissues, reducing off-target toxicity and increasing therapeutic efficacy. Co-encapsulation strategies involving anticancer drugs and resistance inhibitors within nanoparticles have shown potential in achieving coordinated pharmacokinetic and pharmacodynamic profiles. This review discusses the mechanisms of drug resistance, the limitations of current inhibitors, and the advantages of nanoparticle delivery systems in overcoming these challenges. By advancing these technologies, we can enhance treatment outcomes and move towards more effective cancer therapies.

## 1. Introduction

In current oncology, ongoing efforts to completely eradicate cancer have led to advancements in chemotherapy, targeted therapy, and immunotherapy [1,2,3]. Despite these advancements, a persistent challenge remains, specifically drug resistance [1,4,5,6]. Resistance to the administered drugs reduces their efficacy at standard doses, and increasing the dosage to overcome resistance often results in non-specific toxicity, exacerbating patient suffering [2,7]. Currently, overcoming drug resistance typically involves developing new drugs each time resistance occurs, which is a highly inefficient approach [8,9]. Therefore, addressing drug resistance is a critical area that urgently needs improvement in the next generation of cancer therapeutics [10,11].

To address this issue, it is essential to first understand the mechanisms by which cancer cells acquire drug resistance, as they can develop resistance through various pathways [5,12]. When tumor cells are continuously exposed to anticancer agents, they may acquire resistance by undergoing genetic mutations [8,12,13], overexpressing specific proteins [5,14], or hyperactivating drug efflux pumps [15,16]. For instance, cancer cells can recognize apoptotic signals induced by chemotherapeutic agents and overexpress proteins such as Bcl-2, IAP, and Akt to inhibit apoptosis [17,18], or they can overexpress proteins like P-gp to eject the drugs from the cell, resulting in reduced efficacy [19,20]. In addition to these mechanisms, there are various other pathways that contribute to drug resistance, and numerous studies are currently being conducted to analyze and address these issues [14]. Based on these analyses, a range of drugs have been developed to inhibit these resistance mechanisms; however, new side effects have emerged as a result [10,12].

To provide further detail, a range of drugs have been developed to inhibit overexpressed Bcl-2, IAP, Akt, and P-gp, including the following [18,21,22]. Notable examples include navitoclax (Bcl-2 inhibitor), SMAC mimetics (IAP inhibitor), ipatasertib (Akt inhibitor), and tariquidar (P-gp inhibitor), which inhibit these proteins by binding to their active sites, inducing proteasomal degradation, or inhibiting phosphorylation activity [23,24,25,26]. However, these drugs often exist as small molecules or peptide-based agents mimicking proteins, resulting in suboptimal pharmacokinetic/pharmacodynamic (PK/PD) properties and low bioavailability [10,27]. Consequently, these drugs can exhibit off-target toxicity, causing issues similar to those of traditional chemotherapeutics, such as hepatotoxicity, skin rash, and gastrointestinal complications [28]. Therefore, it is crucial to develop effective drug delivery platforms that can safely target these drugs to the desired sites of action [29,30,31].

A drug delivery strategy utilizing nanostructures can be an excellent solution to address these issues [32,33,34]. Nanostructures of sizes from 10 nm to 200 nm can passively target and accumulate in tumors through the EPR effect [35,36]. Furthermore, to optimize the EPR effect, nanoparticles can be utilized to regulate the tumor microenvironment (TME), including tumor blood vessels and blood flow [37,38]. Various factors within the TME, such as the extracellular matrix (ECM), tumor cell density, hypoxia, and interstitial fluid pressure, contribute to the heterogeneity of EPR-based tumor targeting responses. These pathophysiological factors are crucial considerations in developing personalized and improved nanodrug treatments using the EPR effect [35,39]. With these considerations, various nanocarriers, such as polymeric, lipid-based, peptide-based, and inorganic nanoparticles, have been developed and used according to their unique properties, demonstrating improved drug delivery efficiency [40,41,42,43,44,45]. By employing these nanocarriers to encapsulate resistance inhibitors, it is possible to minimize off-target toxicity, enhance tumor-targeting efficiency, and significantly improve drug efficacy, while also potentially reducing the required drug dosage [44]. Therefore, applying suitable drug delivery platforms to existing resistance inhibitors is of critical importance [46].

**Scheme 1:**
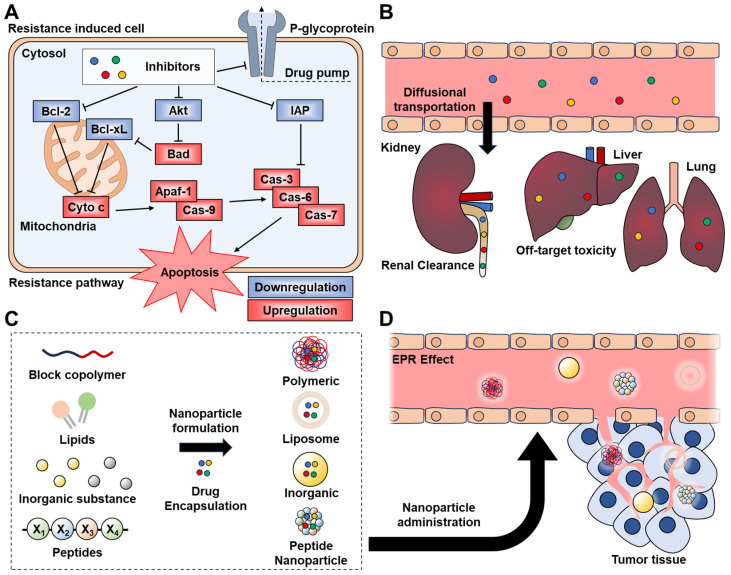
Schematic illustration of the delivery strategies for drug resistance inhibitors to resistance-induced tumor cells. (**A**) Resistance inhibition mechanisms of Bcl-2, IAP, Akt, and P-gp inhibitors. (**B**) Challenges of traditional inhibitors, such as renal clearance and off-target toxicity. (**C**) Drug-encapsulated nanoparticle formulations with block copolymers, lipids, inorganic substances, and peptides. (**D**) Tumor accumulation of nanoparticles via the EPR effect.

Herein, this review presents various strategies for delivering different drug resistance inhibitors encapsulated in nanostructures to enhance therapeutic efficacy while reducing side effects (Scheme 1). Inhibitors for Bcl-2, IAP, Akt, and P-gp were encapsulated in nanocarriers such as polymeric nanoparticles, liposomes, inorganic nanoparticles, and peptide-derived nanoparticles (Table 1). Some of the proposed strategies involve co-encapsulating anticancer drugs and resistance inhibitors within a single nanoparticle to evaluate their interactions and demonstrate coordinated PK/PD characteristics [47,48,49,50,51]. By outlining these strategies, this review aims to provide an effective guide for the future delivery of drug resistance inhibitors using drug delivery platforms. 

## 2. Nano-Delivery of Bcl-2 Inhibitor

Bcl-2 is a pivotal regulator of apoptosis, often overexpressed in various cancers, including chronic lymphocytic leukemia (CLL), diffuse large B-cell lymphoma (DLBCL), breast cancer, and lung cancer, allowing cancer cells to evade apoptosis, leading to chemoresistance and poor prognosis [63,64,65]. Mechanistically, Bcl-2 binds to pro-apoptotic proteins such as Bcl-2-associated X protein (Bax) and Bcl-2 homologous antagonist/killer (Bak), preventing them from oligomerizing and permeabilizing the mitochondrial outer membrane, effectively blocking the release of cytochrome c and the subsequent activation of the caspase cascade [66,67,68,69]. Clinically, Bcl-2 inhibitors such as venetoclax, navitoclax, and obatoclax have demonstrated potential in targeting this pathway and are currently in use or under investigation [70,71,72,73]. Despite their potential, Bcl-2 inhibitors face significant challenges such as resistance development, systemic toxicity, and poor bioavailability, prompting extensive research into nanoparticle-based delivery methods to overcome these limitations [74,75]. Utilizing nanoparticle delivery systems can mitigate these challenges by enhancing drug solubility, stability, and targeted delivery, thereby optimizing the therapeutic efficacy and safety of Bcl-2 inhibitors [76,77].

**Figure 1:**
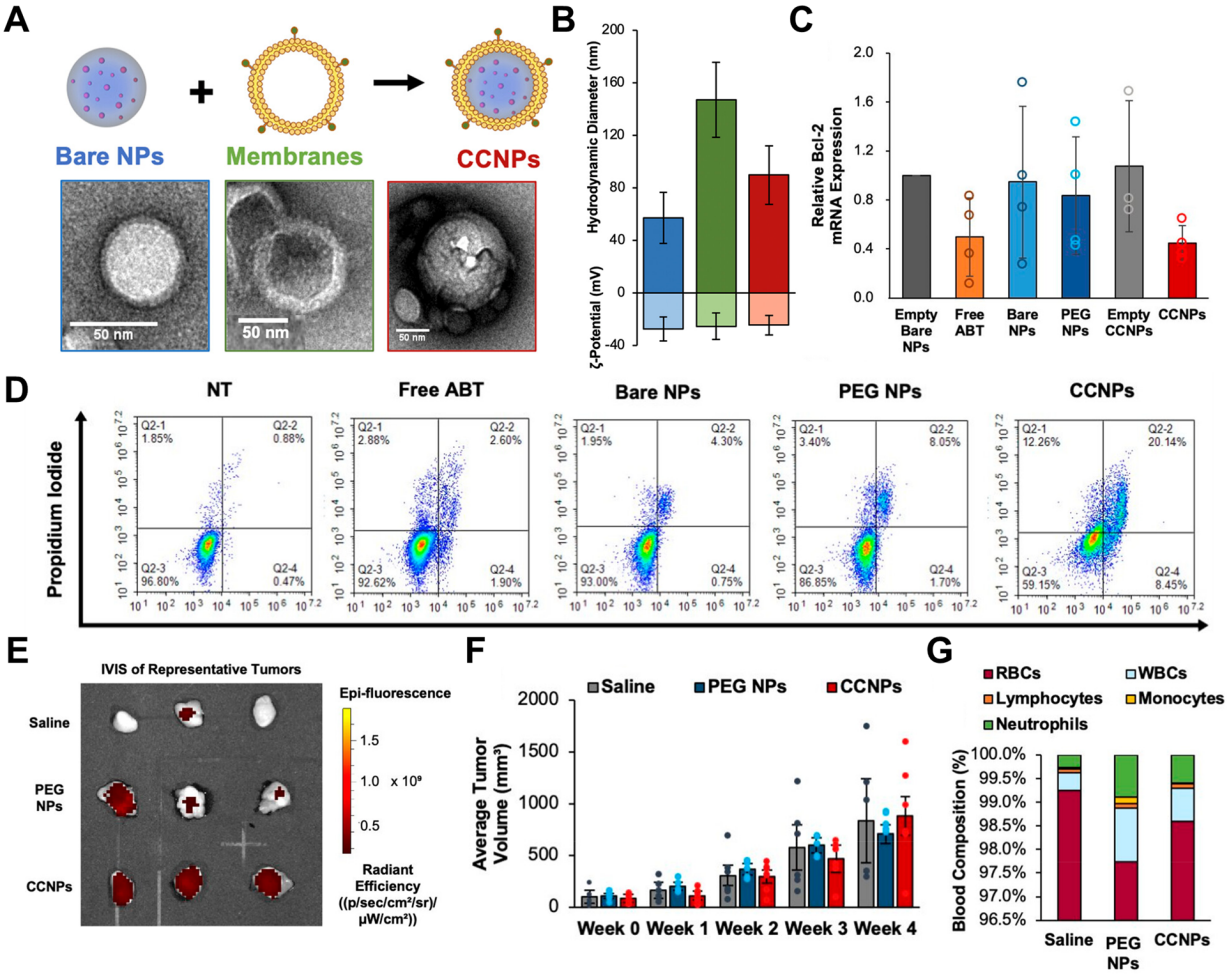
(**A**) Schematic diagram and transmission electron microscopy (TEM) images of ABT-loaded PLGA nanoparticles (NPs), 4T1 cell membrane vesicles, and ABT-coated cell membrane nanoparticles (CCNPs). (**B**) Dynamic light scattering (DLS) analysis and TEM images showing the hydrodynamic diameter and zeta potential of bare NPs, empty membrane vesicles, and CCNPs. (**C**) Measurement of Bcl-2 mRNA levels in 4T1 cancer cells treated with 7 μM free ABT or ABT-loaded NPs for 48 h, showing a significant reduction in Bcl-2 mRNA levels with ABT-loaded CCNPs. (**D**) Apoptosis analysis in 4T1 cells treated with 7 μM ABT, either free or within NPs, after 24 or 48 h, using flow cytometry with Annexin V-FITC and PI staining, demonstrating effective apoptosis induction by ABT-loaded CCNPs. (**E**) Comparison of tumor accumulation of DiD-loaded PEG NPs and CCNPs in an orthotopic murine mammary fat pad tumor model, quantified by tissue weight and tumor radiant efficiency per tumor mass. (**F**) Kaplan-Meier survival curves for mice treated with saline, ABT-loaded PEG NPs, or ABT-loaded CCNPs, showing a modest reduction in tumor burden with ABT-loaded CCNPs. (**G**) Analysis of blood composition post-treatment, showing red and white blood cell counts in mice treated with saline, ABT-loaded PEG NPs, or ABT-loaded CCNPs, indicating reduced systemic toxicity with ABT-loaded CCNPs. Reproduced with permission [52]. Copyright 2023, ACS publications.

M. A. Scully et al. developed a novel nanoplatform utilizing cancer cell membrane-wrapped poly(lactic-co-glycolic acid) (PLGA) nanoparticles (CCNPs) for the targeted delivery of the Bcl-2 inhibitor ABT-737 [52]. The synthesis involved preparing ABT-loaded PLGA nanoparticles, which were then coated with extruded 4T1 cell membrane vesicles to form ABT CCNPs. Transmission electron micrographs validated the core-shell structure, showing successful wrapping of PLGA cores with 4T1 cell membranes (Figure 1A). Dynamic light scattering (DLS) analysis indicated minimal size changes post-coating (Figure 1B). The therapeutic potential of CCNPs was evaluated by measuring Bcl-2 mRNA and protein expression in TNBC cells treated with 7 μM ABT (free or in NPs) for 48 h. Results showed a 55% reduction in Bcl-2 mRNA expression with ABT-loaded CCNPs, similar to free ABT, and significantly higher than bare NPs and PEG NPs (Figure 1C). Flow cytometry analyses confirmed that ABT CCNPs induced apoptosis in 4T1 cells after 24 or 48 h of treatment, with varying levels of early and late apoptosis, and necrosis (Figure 1D). The in vivo efficacy and safety of ABT-loaded CCNPs were tested using an orthotopic murine mammary fat pad tumor model. Female Balb/cJ mice with 4T1 tumors received intravenous injections of DiD-loaded PEG NPs or CCNPs. CCNPs demonstrated significantly higher tumor accumulation compared to PEG NPs (Figure 1E). Kaplan−Meier survival curves indicated modest tumor burden reduction with ABT-loaded CCNPs in the first three weeks, diminishing by the fourth week post-treatment (Figure 1F). Blood samples showed that ABT-loaded PEG NPs reduced red blood cell counts and increased white blood cell counts, indicating inherent drug toxicity. In contrast, ABT-loaded CCNPs did not adversely affect blood composition or show signs of thrombocytopenia (Figure 1G).

A gold nanoparticle (AuNP)-based delivery system to co-deliver Bcl-2 siRNA and doxorubicin (DOX) for more effective cancer therapy was developed by C. Ü. Tunç and colleagues [53]. This novel approach combines gene silencing and chemotherapy to enhance therapeutic outcomes. The synthesis involved using 13 nm AuNP with Bcl-2 siRNA attached to the AuNP surface and DOX intercalated directly to the siRNA, creating a stable and multifunctional delivery system. AuNPs are highly suitable for therapeutic delivery due to their high biocompatibility, low toxicity, and easy surface functionalization, which allows them to carry both gene therapies and chemotherapeutic agents. Their small size and high surface area-to-volume ratio enable a high drug loading capacity, and their stability ensures controlled release in the body. The system’s efficacy was evaluated through various experiments. Successful loading of Bcl-2 siRNA and DOX was confirmed using UV/Vis spectroscopy and dynamic light scattering (DLS) analysis, which showed increased nanoparticle size at each modification stage. In vitro studies using triple-negative breast cancer (TNBC) cells demonstrated effective absorption of the nanoparticles. Dox-loaded nanoparticles significantly reduced cell viability compared to free DOX. Co-delivery of Bcl-2 siRNA and DOX decreased Bcl-2 protein expression by 55%, more effective than single-agent treatments. Flow cytometry confirmed higher apoptosis rates in TNBC cells treated with the DOX-Bcl2-AuNPs system. In vivo efficacy and safety were assessed using a mouse model with implanted tumors. The AuNPs showed high tumor accumulation, indicating targeted delivery. Kaplan-Meier survival analysis revealed significantly extended survival in treated mice. Blood analysis indicated stable red and white blood cell counts, suggesting reduced systemic toxicity. Morphological studies showed significant changes in TNBC cell morphology after treatment, with Bcl-2 siRNA and DOX causing cell contraction and reduced adhesion, indicative of apoptosis. Similar results were observed in MCF7 cells. 

N. Mehrotra and colleagues have pioneered the development of a polylactic acid (PLA)-based polymeric nanoparticle system designed to co-deliver navitoclax and decitabine, presenting a promising approach to overcoming the limitations of conventional chemotherapy such as poor solubility, unsynchronized pharmacokinetics, and disproportionate tumor accumulation [54]. By employing nanoparticles, this strategy enhances drug stability, bioavailability, and therapeutic efficacy while mitigating dose-related toxicity. Navitoclax, a BH3 mimetic and pan-Bcl-2 inhibitor, demonstrates potent activity against hematologic malignancies and some solid tumors. However, one of the significant challenges with navitoclax is its dose-limiting thrombocytopenia, resulting from Bcl-XL inhibition. This adverse effect increases bleeding risk, compromises patient safety, and limits the therapeutic consistency and effectiveness of the treatment. Consequently, thrombocytopenia significantly restricts the use of navitoclax, particularly in combination therapies. Decitabine, a DNA methyltransferase inhibitor, is FDA-approved for treating hematologic malignancies but has limited success as a monotherapy for solid tumors due to its poor stability and short half-life. Combining these two drugs in a single nanoparticle system aims to leverage their synergistic potential, providing a more robust therapeutic outcome. The PLA-based hybrid block copolymeric nanoparticles were synthesized using methoxy polyethylene glycol (mPEG) and Pluronic L-61 as macroinitiators through ring-opening polymerization. PLA-based nanoparticles are ideal for drug delivery due to their biocompatibility, biodegradability, and ease of drug release control. Navitoclax and decitabine were encapsulated using a double emulsion solvent evaporation method, achieving high encapsulation efficiencies (99% for navitoclax and up to 70% for decitabine) and controlled release profiles. The nanoparticles displayed a hydrodynamic diameter ranging from 90 to 145 nm and a surface charge from −18.8 to −11.2 mV. In cellular studies, the dual drug-loaded nanoparticles exhibited significant synergistic cytotoxic effects on breast cancer and acute myeloid leukemia (AML) cell lines. Confocal laser scanning microscopy and flow cytometry confirmed that the nanoparticles were efficiently internalized by the cancer cells, with navitoclax-loaded nanoparticles showing strong colocalization with mitochondria, indicating effective targeting of the intracellular action site. In tumor-bearing mouse models, the dual drug-loaded nanoparticles demonstrated high accumulation in tumor tissues and maintained detectable drug levels in plasma even after 48 h. The nanoparticles exhibited excellent hemocompatibility, significantly reducing platelet toxicity compared to free navitoclax. This reduction in thrombocytopenia risk enhances the therapeutic index, allowing for more consistent and effective treatment. Tumor growth inhibition studies in AML and breast cancer models showed that the dual drug-loaded nanoparticles effectively suppressed tumor growth, with notable reductions in tumor volume and extended survival times in treated mice. Further toxicity evaluations revealed that the nanoparticles exhibited low protein adsorption in blood, minimizing interference with systemic circulation. Additionally, hemolysis tests indicated that the nanoparticles were suitable for intravenous administration, displaying low hemolytic activity. 

In summary, drug delivery strategies have been developed to accurately deliver Bcl-2 inhibitors, which play a crucial role in apoptosis and induced drug resistance, to target sites. These drug carriers include polymeric nanoparticles and AuNPs, and these drug delivery strategies can effectively enhance the efficacy of existing inhibitors to suppress drug resistance. This drug delivery technology is expected to serve as a milestone for the delivery methods of future Bcl-2 inhibitors. 

## 3. Nano-Delivery of IAPs Inhibitor

IAPs are critical regulators of apoptosis, often overexpressed in cancers such as melanoma, lung cancer, and pancreatic cancer, where they contribute to therapeutic resistance and poor prognosis [78,79,80]. Mechanistically, IAPs inhibit apoptosis by binding to and blocking caspases, thereby preventing the activation of the apoptotic cascade and promoting cancer cell survival [81,82,83]. Clinically, IAP inhibitors like birinapant and LCL161, which are both second mitochondria-derived activator of caspases (SMAC) mimetics and small molecules, have demonstrated potential in targeting this pathway by mimicking the action of endogenous SMAC, promoting the degradation of IAPs, and restoring apoptosis [84,85,86]. However, their clinical use is often hindered by severe toxicity, such as hepatotoxicity, and limited efficacy when used as monotherapies [87]. Therefore, using nanoparticle delivery systems can address these challenges by directly targeting cancer cells, reducing systemic toxicity, and improving drug stability [88,89].

**Figure 2:**
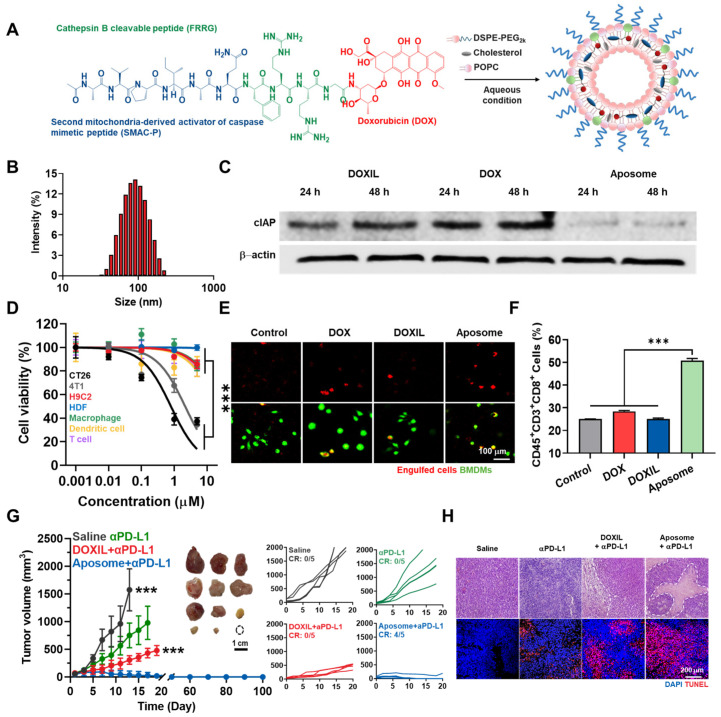
(**A**) Diagram of the SMAC-P-FRRG-DOX prodrug designed for cancer cell specificity and apoptosis induction, formulated into PEGylated liposomes known as Aposomes. (**B**) Size distribution of Aposomes in saline (1 mg/mL). (**C**) Inhibition of IAP expression in CT26 cells treated with free DOX, DOXIL, or Aposomes for 48 h, with statistical analysis using the Tukey-Kramer post-hoc test. (**D**) Viability of CT26 and 4T1 cancer cells, H9C2 and HDF normal cells, and immune cells (M0 and M1 macrophages, dendritic cells, and T cells) after 48 h of treatment with Aposomes. (**E**) Fluorescence images of CellTracker Green-labeled BMDMs co-cultured with CT26 cells treated with free DOX, DOXIL, or Aposomes (pHrodo labeled) for 2 h. (**F**) Proportion of cytotoxic T cells (CD45+CD3+CD8+) among lymphocytes following 48-h co-culture with CT26 cells treated with free DOX, DOXIL, or Aposomes. (**G**) Evaluation of Aposomes combined with immune checkpoint blockade (ICB) in colon tumor models: tumor growth curves in CT26 tumor-bearing mice treated with DOXIL plus αPD-L1 antibody (Ab), Aposomes plus αPD-L1 Ab, or αPD-L1 Ab alone. Both Aposomes and DOXIL were administered at a DOX dose of 3 mg/kg every three days, with αPD-L1 Ab (10 mg/kg) given simultaneously via tail vein injection. (**H**) Tumor tissue analysis using TUNEL and H&E staining after 13 days of treatment. Statistical significance was determined by one-way ANOVA with Dunnett’s test, *** *p* < 0.001). Reproduced with permission [55]. Copyright 2024, BMC.

J. Kim et al. have developed an innovative nanoparticle system termed “Aposomes” to co-deliver SMAC peptide (SMAC-P) and DOX for enhanced cancer therapy [55]. This system aims to exploit the synergistic effects of SMAC-P and DOX to induce potent immunogenic cell death (ICD) and improve immune checkpoint blockade (ICB) therapy efficacy. The researchers synthesized a SMAC-P-FRRG-DOX prodrug, which was then encapsulated into PEGylated liposomes to form Aposomes (Figure 2A). SMAC-FRRG-DOX is developed by conjugating a pro-apoptotic peptide (SMAC; Ala-Val-Pro-Ile-Ala-Gln, AVPIAQ) with a cathepsin B-cleavable peptide (Phe-Arg-Arg-Gly, FRRG) and doxorubicin (DOX). Cathepsin B, a cysteine protease predominantly found in lysosomes, is involved in protein degradation and autophagy and is overexpressed in various cancers such as colorectal, breast, and prostate cancer [90]. In cancer cells, overexpressed cathepsin B specifically cleaves the -FRRG- part of SMAC-FRRG-DOX, releasing DOX. The released DOX accumulates in the nucleus of cancer cells, where it inhibits DNA replication and induces cell death. In contrast, normal cells experience minimal toxicity due to their low levels of cathepsin B. Aposomes exploit this release mechanism by being broken down by cathepsin B into SMAC (AVPIAQFR) and free DOX. This leads to effective apoptosis in cancer cells due to the combined effects of SMAC and DOX. In normal cells, the low expression of cathepsin B results in significantly reduced toxicity. Liposomes play a crucial role as a drug delivery system due to their biocompatibility and biodegradability, which enhance drug stability and protect the drugs until they reach the target cells. The Aposomes exhibited a stable structure with an average diameter of 109.1 nm, making them suitable for effective tumor accumulation through the EPR effect (Figure 2B). The encapsulation process ensured high drug loading efficiency, stability under physiological conditions, and controlled release of the prodrug in response to cathepsin B, an enzyme overexpressed in cancer cells. The team treated CT26 colon cancer cells with DOXIL, free DOX, and Aposomes, then compared IAP expression levels at 24 and 48 h post-treatment (Figure 2C). The results showed that Aposomes released SMAC-P within cancer cells, antagonizing IAPs and sensitizing the cells to DOX-induced apoptosis, resulting in significant cytotoxicity. However, due to the controlled release mechanism mediated by cathepsin B, Aposomes were safe for immune cells such as macrophages, dendritic cells, T cells, and normal cells like HDF and H9C2 (Figure 2D). The combination of SMAC-P and DOX in Aposomes induced higher levels of damage associated molecular patterns (DAMPs) compared to free DOX or DOXIL, thereby enhancing ICD potential and stimulating macrophage activation and T cell responses, creating an immune-responsive tumor microenvironment (Figure 2E,F). In tumor-bearing mouse models, Aposomes demonstrated superior tumor targeting and retention compared to free DOX, leading to higher intratumoral drug concentrations and reduced systemic exposure. This targeting efficiency translated into significant tumor growth inhibition and improved survival rates. Mice treated with a combination of Aposomes and programmed cell death ligand 1 antibodies (anti-PD-L1) showed high rates of complete tumor regression and prevention of recurrence (Figure 2G), with excellent apoptosis observed through TUNEL staining (Figure 2H). This combined approach underscores the potential of enhancing cancer immunotherapy.

T. Wang and colleagues developed matrix metalloproteinase (MMP)-responsive Poly(vinyl alcohol) (PVA)-peptide conjugates (PPCs) to enhance immune checkpoint therapy by co-delivering IAP antagonists [56]. These PPCs reassemble within the tumor microenvironment (TME), increasing PD-L1 occupancy and blocking efficiency. The PPC nanoparticles, synthesized through a Michael-type addition reaction, had an average size of 110 nm and remained stable under physiological conditions. Upon exposure to MMP-2, PPC-1 transformed into β-sheet fibrous structures, enhancing their stability and drug delivery efficiency. In experiments with B16F10 cells, PPC-1 formed nanofibers in response to MMP-2, reducing cellular uptake, whereas PPC-2 remained spherical and was internalized by the cells. PPC-1 showed a higher programmed cell death 1 (PD-1)/PD-L1 blocking effect, achieving a 50–60% blocking rate compared to PPC-2’s 30%. The IAP antagonist AZD5582 activated dendritic cells (DCs), increasing the expression of maturation markers CD80 and CD86 and elevating the secretion of cytokines such as tumor necrosis factor-α (TNF-α) and interleukin-12p40 (IL-12p40), demonstrating potent immunomodulatory effects. In B16F10 tumor-bearing mice, PPC-1 showed enhanced tumor accumulation and retention, with fluorescence intensity 1.5 times higher than PPC-2, remaining stable for up to 96 h. The combination of PPC-1 and AZD5582 significantly inhibited tumor growth and improved survival rates, showing an 80% tumor inhibition rate compared to 58% for PPC-2 with AZD5582. Immunohistochemical analysis revealed decreased Ki67 expression and increased TUNEL staining, indicating reduced tumor cell proliferation and enhanced apoptosis. The combination treatment of PPC-1 and AZD5582 increased CD8+ T cell infiltration and reduced Treg cells in the TME, significantly improving the CD8+/Treg ratio. In a tumor recurrence model, this combination therapy inhibited secondary tumor growth and increased the percentage of memory T cells (CD8+CD44+), indicating the establishment of immune memory. In the Pan02 pancreatic cancer model, the PPC-1 and AZD5582 combination therapy similarly inhibited tumor growth and enhanced immune response, increasing the CD8+/Treg ratio and memory T cells. 

C. M. Lin et al. developed a novel system using hyaluronic acid (HA)-coated AuNP (AuNP-HA) to deliver IAP-2-specific siRNA, inhibiting oncogenic properties in lung cancer A549 cells induced by benzo[a]pyrene (BaP) [57]. HA targets CD44-overexpressed cancer cells, ensuring specific delivery and minimizing off-target effects. AuNPs provide excellent biocompatibility and ease of surface modification, enhancing drug stability and controlled release. The synthesis of AuNP-HA involved coating gold nanoparticles with HA, confirmed through UV-Vis spectrophotometry and FTIR. Cell viability assays showed no significant cytotoxicity, demonstrating biocompatibility. Internalization studies revealed efficient uptake of AuNP-HA by A549 cells via CD44-mediated endocytosis. Knocking down CD44 significantly reduced uptake, confirming its role in internalization. Therapeutic efficacy was evaluated in BaP-challenged A549 cells. AuNP-HA-IAP-2 siRNA significantly reduced cell proliferation and induced apoptosis, evidenced by increased Annexin V-FITC and PI staining. Western blot analyses showed decreased anti-apoptotic proteins IAP-2 and Bcl-2 and increased pro-apoptotic proteins Bax and active caspase-3. Moreover, AuNP-HA-IAP-2 siRNA treatment significantly inhibited BaP-induced cell motility and invasiveness, primarily through the suppression of MMP-2 activity. This highlights the system’s efficacy in hindering cancer cell migration and invasion.

In short, IAP, which inhibits the activation of the apoptotic cascade by binding to caspase, is commonly overexpressed in cancer cells and induces drug resistance. Although various drugs have been developed to inhibit IAP, they have shown limitations such as low PK, leading to extensive research on developing nanoparticle-based delivery strategies. IAP inhibitors have been effectively delivered to tumor sites using liposomes, polymeric nanoparticles, and AuNPs. Based on these studies, future research on delivering IAP inhibitors to tumor sites is expected to be active. Additionally, as seen in the study by J. Kim et al., other drugs can also be encapsulated to achieve synergistic effects without any concerns of activating IAPs [55].

## 4. Nano-Delivery of Akt Inhibitor

Akt (Protein Kinase B) is a serine/threonine kinase that plays a crucial role in cell survival, growth, and metabolism, and is frequently overactivated in cancers such as breast, prostate, and lung cancers [91,92]. Overexpressed Akt promotes cell survival and proliferation by phosphorylating and inhibiting components of the apoptotic machinery, such as Bad and caspase-9, and activating survival pathways like mammalian target of rapamycin (mTOR), thereby antagonizing the effects of apoptosis-inducing drugs [93,94]. Abnormal activation of Akt can result from both inherited mutations and acquired changes, particularly manifesting as a challenge after cancer treatment [24,95,96]. Clinically, Akt inhibitors like perifosine, ipatasertib, and MK-2206 have been evaluated; however, they are limited by significant side effects and therapeutic constraints [97,98,99,100]. Utilizing nanoparticle delivery systems can address these challenges by enhancing drug solubility, improving stability, and ensuring targeted delivery to cancer cells, thereby enhancing the therapeutic efficacy and safety profile of Akt inhibitors [101,102,103].

**Figure 3:**
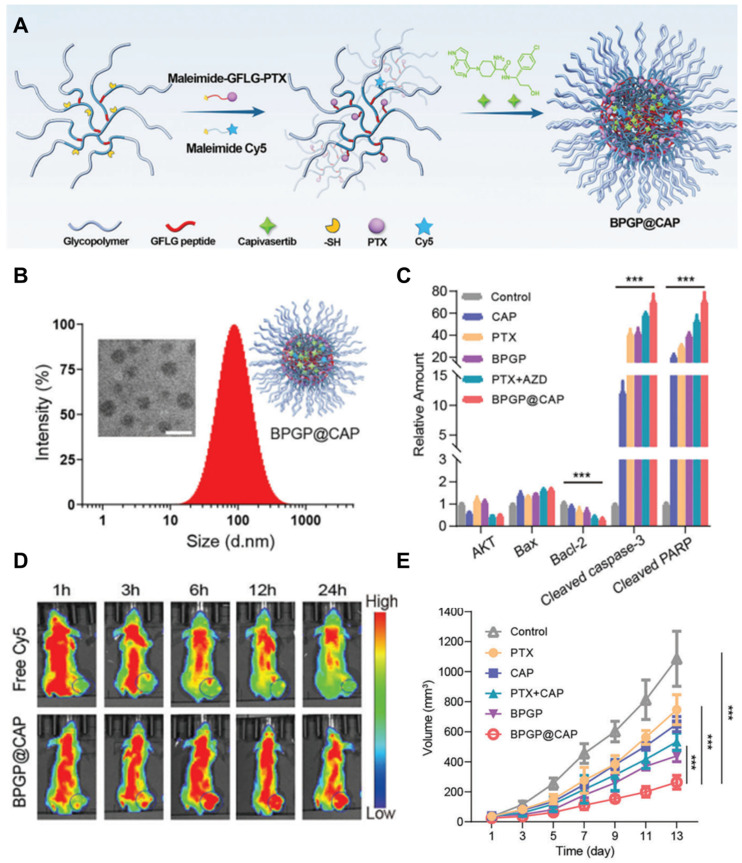
(**A**) Schematic illustration of enzyme-responsive branched glycopolymer-based nanoassembly (BPGP@CAP) for synergistic antitumor therapy. A branched poly(LAEMA)-GFLG-PTX prodrug self-assembles through hydrophobic-hydrophilic interactions and this prodrug encapsulates CAP as an AKT inhibitor to form a stable nanoassembly, BPGP@CAP. (**B**) Hydrodynamic diameters and representative TEM images of BPGP and BPGP@CAP, respectively. Scale bars = 100 nm. (**C**) Western blot results of proteins associated with apoptosis in MFC cells after exposure to PTX, CAP, PTX + CAP, BPGP, and BPGP@CAP, including Bax, Bcl-2, cleaved-caspase-3 and cleaved PARP. The proteins in the control group are significantly lower than those in the groups treated with PTX, CAP, PTX + CAP, BPGP, and BPGP@CAP, and the *p*-values are less than 0.001. (**D**) Biodistribution and tumor growth inhibition of BPGP@CAP in vivo. Fluorescent images of the mice with MFC tumors exposed to Cy5-labelled BPGP@CAP and free Cy5 at different time-points. (**E**) Average tumor growth curves in different treatment groups (*n* = 5). Statistical significance was determined by two-sided unpaired Student’s *t*-test, *** *p* < 0.001). Reproduced with permission [58]. Copyright 2024, Wiley-VCH.

X. Song and colleagues developed an innovative nano-delivery system to overcome drug resistance by co-delivering Akt pathway inhibitors and the chemotherapy agent paclitaxel (PTX) [58]. This system uses enzyme-responsive glycopolymer-based nano-assemblies to enable precise drug delivery and controlled release within the tumor microenvironment. Utilizing enzyme-responsive glycopolymer-based nanoassemblies, this approach facilitates precise drug delivery and controlled release within the tumor microenvironment. Enzymes commonly employed in such systems include cathepsin B, cathepsin L, alkaline phosphatase, and MMP2. These enzymes, overexpressed in specific tumor microenvironments, promote the disassembly of nanoassemblies and regulate drug release. In this study, the system is rapidly broken down by the overexpressed cathepsin B enzyme in lysosomes, leading to the release of PTX and CAP [104,105]. This approach offers higher stability and specificity compared to traditional nano-delivery systems and can play a crucial role in overcoming drug resistance. A branched poly(LAEMA)-GFLG-PTX (BPGP) prodrug self-assembles through hydrophobic-hydrophilic interactions, and this prodrug encapsulates capivasertib (CAP) as an Akt inhibitor to form a stable nano-assembly, BPGP@CAP. This nano-assembly combines enzyme responsiveness and tumor targeting, becoming selectively activated within the tumor site (Figure 3A). The physicochemical properties of BPGP and BPGP@CAP are demonstrated through their hydrodynamic diameters and transmission electron microscope (TEM) images. Both BPGP and BPGP@CAP exhibit uniform size distributions and maintain stable structures (Figure 3B). This stability ensures safe delivery to the tumor site. Western blot analysis shows that CAP inhibits the expression of proteins related to the phosphoinositide 3-kinases (PI3K)/Akt pathway. After CAP treatment, the expression of Bax, cleaved caspase-3, and cleaved PARP increased in MFC cells, indicating apoptosis induction following Akt inhibition (Figure 3C). These results highlight the effective suppression of tumor cell survival by the combination of PTX and CAP. The in vivo distribution of BPGP@CAP is confirmed through fluorescent images from mice injected with Cy5-labeled BPGP@CAP and free Cy5. BPGP@CAP selectively accumulates in tumor sites, suggesting that the nano-assembly accumulates at high concentrations in tumor sites, enhancing therapeutic efficacy (Figure 3D). This selective accumulation improves the precision of drug delivery and minimizes side effects on normal cells. The antitumor effect of BPGP@CAP is demonstrated by the significant inhibition of tumor growth in mice treated with BPGP@CAP. The combination of PTX and CAP is shown to be more effective than single-agent treatments, with measurements of tumor volume and inhibition rate confirming the high antitumor efficacy of BPGP@CAP (Figure 3E). These results emphasize the synergistic effect of PTX and CAP and the crucial role of the nano-delivery system in overcoming drug resistance in tumor treatment. 

By utilizing a nano-delivery system for Akt inhibition, T. Zuo et al. developed a novel multistage acidity and cathepsin B (CatB) enzyme-responsive nanocarrier for the co-delivery of Akt pathway inhibitors and chemotherapy drugs [59]. These nanocarriers (PPP-DA/NPs) are designed to enhance drug delivery efficiency, improve cellular uptake, and achieve precise tumor targeting by leveraging the pH and enzyme characteristics of the tumor microenvironment. The nanocarriers consist of a core containing the chemotherapeutic agent docetaxel (DTX) and the PI3K/Akt inhibitor GDC0941, encapsulated in an outer shell modified with 2,3-dimethylmaleic anhydride (DA) for pH-sensitive charge reversal and enzyme-responsive drug release. Characterization using DLS and TEM showed that PPP-DA/NPs have a uniform spherical morphology with an average particle size of 135.6 nm, maintaining stability under physiological conditions. This system distinguishes itself from traditional PLGA nanoparticles by releasing drugs in response to acidic conditions and CatB enzymes in the tumor microenvironment. To verify the efficacy of PPP-DA/NPs in inducing apoptosis, MTT assays were performed on breast cancer cells. The results indicated that PPP-DA/NPs induced significant apoptosis in 4T1 cells, demonstrating greater efficacy than free DTX and GDC0941. Western blot analysis was conducted to assess the impact on the PI3K/Akt pathway, revealing that PPP-DA/NPs effectively inhibited the pathway by reducing phosphorylation levels of Akt. These findings underscore the effective suppression of tumor cell survival through the synergistic action of DTX and CAP. In a mouse model of breast cancer, administration of PPP-DA/NPs resulted in a marked suppression of tumor growth and metastasis. Treated mice exhibited significantly reduced tumor volumes, underscoring the nanocarrier’s potential in overcoming drug resistance and enhancing therapeutic outcomes.

J. Gonzalez-Valdivieso and colleagues developed an innovative nanodelivery system to overcome drug resistance in pancreatic cancer treatment [60]. This system focuses on using self-assembling genetically engineered polymeric nanoparticles formed by elastin-like recombinamers (ELRs) to deliver a small peptide inhibitor of protein kinase Akt to pancreatic cancer cells. The research team explored how ELR-based nanoparticles could deliver an Akt inhibitor to target the overexpressed Akt kinase in pancreatic cancer cells. ELR-based nanoparticles self-assemble into monodisperse particles of approximately 73.1 nm, capable of inhibiting the phosphorylation and activation of the Akt protein, blocking the NF-κB signaling pathway, and triggering caspase 3-mediated apoptosis. Using two clinically relevant pancreatic cancer patient-derived cell lines, PDX185 and PDX354, the efficacy of ELR nanoparticles was evaluated. The nanoparticles were effectively internalized by the cancer cells and localized within lysosomes, as confirmed by cellular uptake studies using flow cytometry and confocal microscopy. This lysosomal localization is crucial for the proper release of the Akt inhibitor, as the cathepsin D-sensitive sequence included in the ELRs is recognized by lysosomal proteases, facilitating the release of the inhibitor into the cytoplasm. Metabolic activity and cell viability assays demonstrated that ELR nanoparticles carrying the Akt inhibitor significantly reduced the metabolic activity and viability of pancreatic cancer cells in a dose- and time-dependent manner. Control nanoparticles without the inhibitor did not affect cell viability, confirming their biocompatibility. The treatment led to increased expression of apoptosis-related proteins such as cleaved caspase-3 and cleaved PARP, indicating the induction of apoptosis in the cancer cells.

In conclusion, Akt is a protein that plays a crucial role in cell proliferation, and its overexpression activates the cell survival pathway, increasing drug resistance. To inhibit this overexpression of Akt, various drugs have been developed, and to maximize the efficacy of these developed drugs, various targeted delivery methods using drug carriers such as polymeric and polypeptide nanoparticles have been studied. These studies will serve as a starting point for future research on delivery systems to maximize the efficacy of additional Akt inhibitors and are ultimately expected to effectively inhibit the drug resistance induced by Akt. 

## 5. Nano-Delivery of P-Glycoprotein Inhibitor

P-gp, also known as ATP binding cassette subfamily B member 1 (ABCB1), is a membrane-bound efflux transporter that plays a critical role in the multidrug resistance (MDR) phenomenon observed in many cancers, including breast, ovarian, and colorectal cancers [16,106,107]. By actively pumping out a wide variety of chemotherapeutic agents from cancer cells, P-gp reduces intracellular drug accumulation, thereby diminishing the efficacy of these treatments and contributing to therapeutic resistance [19,108,109]. The overexpression of P-gp is often driven by genetic and epigenetic changes, which can be both inherited and acquired during the course of cancer treatment [20,110]. Clinically, P-gp inhibitors such as tariquidar, elacridar, and zosuquidar have been investigated to overcome this resistance mechanism; however, their use has been limited by significant challenges, including systemic toxicity and poor bioavailability [111,112,113]. Nanoparticle-based drug delivery systems offer a promising solution to these issues by enhancing drug solubility, stability, and targeted delivery, which can reduce systemic toxicity and improve the therapeutic outcomes of P-gp inhibitors [114,115,116].

**Figure 4:**
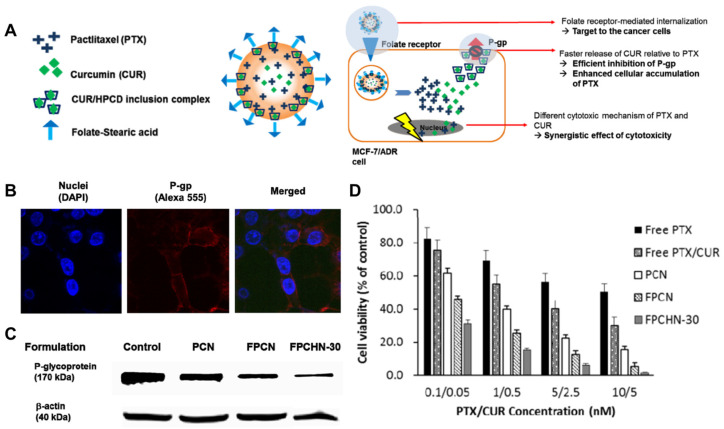
(**A**) Schematic illustration of folate-conjugated paclitaxel and curcumin/HPCD co-loaded lipid nanoparticles (FPCHN-30). (**B**) Expression of P-gp on the surface of MCF-7/ADR cells treated with FPCHN-30 for 4 h, stained with a primary MDR antibody followed by a secondary Alexa 555 antibody. (**C**) Western blot analysis showing P-gp and β-actin expression, and normalized P-gp levels in MCF-7/ADR cells treated with PCN, FPCN, and FPCHN-30 for 4 h. (**D**) Cytotoxicity of different formulations (PCN, FPCN, and FPCHN-30) in MCF-7/ADR cells after 48 h (*n* = 3, mean ± SD). Reproduced with permission [61]. Copyright 2017, Impact Journals, LLC.

To address P-gp inhibition, nanoparticle-based drug delivery systems have recently been developed by Baek et al. [61]. They developed FPCHN-30 nanoparticles which incorporated curcumin as a 2-hydroxypropyl-β-cyclodextrin (HPCD) inclusion complex, enabling its faster release compared to paclitaxel (Figure 4A). This sequential release effectively inhibits P-gp, increasing the intracellular accumulation of paclitaxel. Additionally, FPCHN-30 is designed with folate conjugation, allowing it to selectively bind to cancer cells overexpressing folate receptors. This targeted delivery minimizes side effects on non-target tissues and maximizes therapeutic efficacy. FPCHN-30 suppresses P-gp expression on the surface of MCF-7/ADR cells after 4 h of treatment. P-gp was stained with a primary MDR antibody followed by a secondary Alexa 555 antibody, and the results indicate a significant reduction in P-gp expression with FPCHN-30 treatment (Figure 4B). Western blot analysis of p-gp and β-actin expression in MCF-7/ADR cells treated with PCN, FPCN, and FPCHN-30 for 4 h further confirms the superior P-gp inhibitory ability of FPCHN-30, which positively correlates with the cytotoxicity study results (Figure 4C). The cytotoxicity study results of different formulations on MCF-7/ADR cells over 48 h compared the cytotoxicity of PCN, FPCN, and FPCHN-30, with FPCHN-30 showing the highest cytotoxicity. This difference was statistically significant (* *p* < 0.05; ** *p* < 0.01) (Figure 4D). 

C. G. Zhang and colleagues developed N-succinyl chitosan-poly-L-lysine-palmitic acid (NSC-PLL-PA)-based triblock copolymer micelles to tackle MDR [39]. These micelles co-deliver DOX and P-gp siRNA, effectively inhibiting P-gp expression and allowing drugs to accumulate inside cancer cells. NSC-PLL-PA micelles are biocompatible and biodegradable, minimizing long-term toxicity. They remain stable at neutral pH but become unstable in acidic conditions (pH 5.3), common in tumors, ensuring efficient drug release. Cellular studies showed that DOX-siRNA micelles significantly inhibited P-gp expression in HepG2/ADM cells, enhancing anticancer effects. These micelles simultaneously deliver siRNA and DOX, maximizing drug synergy. Stability tests revealed that DOX-siRNA micelles are stable at pH 7.4 but unstable at pH 5.3, leading to rapid drug release in tumors. Cellular uptake studies demonstrated efficient delivery of DOX and siRNA into HepG2/ADM cells, co-localizing in the cytoplasm for effective P-gp inhibition and anticancer action. Cytotoxicity assays showed increasing DOX-siRNA micelle concentrations led to higher cytotoxicity, with maximum effect at siRNA concentrations around 100 nM. This indicates effective P-gp inhibition, enhancing DOX’s effects. In vivo distribution studies in mice revealed that micelles predominantly accumulated in tumors within 24 h, demonstrating efficient targeting through the EPR effect. 

O. Esim et al. designed PLGA-based nanoparticles for the co-delivery of DOX and verapamil (Ver) to overcome multidrug resistance (MDR) [62]. The researchers encapsulated DOX and Ver in PLGA nanoparticles to inhibit P-gp mediated drug efflux and evaluated their anticancer activity in breast cancer (MCF-7/ADR) cells. The team used a double emulsion solvent evaporation method to create PLGA nanoparticles with an average size of approximately 200 nm and high encapsulation efficiencies for both drugs. Drug release experiments showed an initial burst release followed by sustained release, with the co-encapsulated nanoparticles exhibiting slower release compared to single-drug nanoparticles. This controlled release profile is advantageous for maintaining therapeutic drug levels over an extended period. To confirm the efficacy of this formulation, cytotoxicity tests showed that DOX-Ver PLGA nanoparticles induced early and late apoptosis in MCF-7/ADR cells, resulting in higher cytotoxicity compared to other formulations. Early apoptosis occurs when cells begin to die. During this process, phosphatidylserine moves to the cell surface and can be detected using Annexin V-FITC staining. In MCF-7/ADR cells treated with nanoparticles containing Dox and Ver (Dox-VerNP), the early apoptosis rate was 13.52 ± 0.06%. Late apoptosis occurs when cells suffer severe damage. This stage is characterized by membrane blebbing, and late apoptosis can be detected using propidium iodide (PI) staining. In Dox-VerNP treated cells, the late apoptosis rate was 53.94 ± 0.15%. Cellular uptake studies indicated that co-delivery increased intracellular DOX levels, likely due to Ver inhibiting P-gp, which reduces drug efflux. 

Sifeng Zhu et al. developed a drug delivery system, HT@ER/PTX, targeting CD44 and mitochondria [117]. This nanoparticle effectively delivers the P-gp inhibitor encequidar (ER) and the chemotherapeutic agent paclitaxel (PTX). HT@ER/PTX nanoparticles are synthesized by first creating T@ER/PTX nanoparticles using the cationic polymer TPP-PEG-PDLLA, PTX, and ER. Afterward, hyaluronic acid (HA), the natural ligand for CD44 overexpressed in MDR ABC cell membranes, is added. This modification enhances the tumor cell-targeting ability by enabling specific binding to CD44 receptors. The HT@ER/PTX nanoparticles (ERmolar ratio 1:1) demonstrated excellent P-gp inhibition and targeted mitochondria in MCF-7/PTX cells, inducing apoptosis. Furthermore, in a mouse model with xenografted MCF-7/PTX cells, HT@ER/PTX showed significantly higher anti-tumor efficacy compared to PTX (Taxol^®^). The tumor inhibition rate of HT@ER/PTX was 72.64% ± 4.41%, compared to 32.36% ± 4.09% for Taxol^®^. Mice treated with HT@ER/PTX also exhibited prolonged survival compared to those treated with Taxol^®^. Additionally, HT@ER/PTX not only inhibited the P-gp-mediated removal of toxic lipid peroxidation byproducts caused by the chemotherapeutic drugs, but also increased the expression of mitochondrial dynamics-related proteins. This promoted oxidative stress damage and activated the caspase-3 apoptosis pathway. These findings suggest that co-delivery of anti-tumor drugs and P-gp inhibitors targeting mitochondria could be an effective approach for treating MDR in ABC.

Hayrettin Tonbul et al. aim to develop and evaluate poly(lactic-co-glycolic acid) (PLGA) nanoparticles co-loaded with paclitaxel and elacridar, targeted at transferrin receptors, to concurrently overcome multi-drug resistance and enhance the delivery of anticancer agents [118]. Drug resistance significantly reduces the efficacy of chemotherapy and the overall survival rate of breast cancer patients. P-glycoprotein (P-gp) inhibitors have the potential to address this issue, but systemic use of such inhibitors, like elacridar, is limited due to their side effects and toxicity. The research team utilized nanoprecipitation methods to fabricate PLGA nanoparticles, which were then decorated with transferrin to specifically target cancer cells that tend to overexpress transferrin receptors, leveraging this characteristic for enhanced drug delivery. Following the characterization and drug release experiments, the efficacy of the nanoparticles was assessed in breast cancer EMT6/AR1.0 cells, which exhibit high resistance to paclitaxel and significant P-gp expression. The transferrin-decorated paclitaxel and elacridar co-loaded PLGA nanoparticles displayed an average particle size of 226.9 nm and a zeta potential of −3.9 mV, with high encapsulation efficiencies between 70–76% for both drugs. The presence of transferrin enhanced the uptake of nanoparticles by breast cancer cells, and the combined delivery of paclitaxel and elacridar successfully overcame resistance and enabled cytotoxicity. In conclusion, the simultaneous and targeted delivery of P-gp inhibitors and anticancer drugs loaded in nanoparticles represents a promising approach for cancer therapy. This study contributes significantly to enhancing the effectiveness of cancer treatment.

Consequently, unlike other resistance-related proteins, P-gp pumps drugs out of the cells. Overactivation of P-gp induces drug resistance through its drug transportation mechanism, which disrupts the accumulation of administered drugs. However, it is important to target cancer to inhibit P-gp due to the critical side effects on normal cells. The nano-delivery system of these inhibitors minimizes such side effects and maximizes the drug efficacy, becoming a beacon for future P-gp treatments.

## 6. Conclusions

One of the biggest issues in modern chemotherapy for tumor treatment is the development of drug resistance due to continuous and repeated administration. Continuous exposure of tumor cells to drugs increases the expression of proteins such as Bcl-2, IAP, and Akt, which inhibit apoptosis and accelerate tumor proliferation, or proteins like P-gp, which transport intracellular drugs out of the cells. These side effects lead to a continuous decrease in drug efficacy, creating a vicious cycle where increasing amounts of the drug are required, resulting in more severe side effects. Therefore, many inhibitors have been developed to inhibit the functions of these proteins, but they also have limitations due to their inherent side effects.

To address these issues, as introduced in this review, many researchers have sought to utilize nanostructure-based drug delivery systems. Nanoparticles can passively target tumors via the EPR effect, minimizing off-target toxicity while increasing therapeutic efficacy. Additionally, drug carriers can resolve issues such as drug solubility and stability, which are common problems with most inhibitors. Therefore, using nanoparticles for drug delivery is an excellent strategy to effectively inhibit drug resistance and minimize the associated side effects.

Certainly, these nanoparticles still have problems, such as insufficient tumor accumulation rates and the toxicity of the nanoparticles themselves. However, nanoparticle-based anticancer drugs like Doxil^®^ are already being used in clinical settings, demonstrating their acknowledged efficacy. Moreover, these nanocarriers have the advantage of co-delivering drug resistance inhibitors and tumor treatment drugs, simultaneously suppressing the overexpression of resistance-inducing proteins in real time, thereby maximizing therapeutic efficacy. Thus, the delivery of drug resistance inhibitors using nanoparticles holds vast potential, and the studies introduced in this review will undoubtedly serve as significant milestones in the research of nanoparticle-based delivery systems for resistance inhibitors.

## Figures and Tables

**Table 1:** Summary of inhibitor delivery strategies for resistance-inducing protein.

Type of Resistance	Inhibitors	Type of Nanoparticle	Ref.
Bcl-2	ABT-737	PLGA	[52]
	Bcl-2 siRNA	AuNP	[53]
	Navitoclax	PLA	[54]
IAP	SMAC mimetic peptide (AVPIAQ)	Liposome	[55]
	AZD5582	PVA-peptide	[56]
	IAP-2 siRNA	AuNP	[57]
Akt	Capivasertib	Poly(LAEMA)	[58]
	GDC0941	PLGA-p-PEI-DA	[59]
	Akt inhibitor peptide (AVTDHPDRLWAWERF)	ELR polypeptide	[60]
P-glycoprotein	Curcumin	2-hydroxypropyl-β-cyclodextrin (HPCD)	[61]
	P-glycoprotein siRNA	N-succinyl chitosan–PLL–palmitic acid (NSC–PLL–PA).	[39]
	Verapamil	PLGA	[62]
